# ER Stress in ERp57 Knockout Knee Joint Chondrocytes Induces Osteoarthritic Cartilage Degradation and Osteophyte Formation

**DOI:** 10.3390/ijms23010182

**Published:** 2021-12-24

**Authors:** Yvonne Rellmann, Elco Eidhof, Uwe Hansen, Lutz Fleischhauer, Jonas Vogel, Hauke Clausen-Schaumann, Attila Aszodi, Rita Dreier

**Affiliations:** 1 Institute of Physiological Chemistry and Pathobiochemistry, Waldeyerstraße 15, 48149 Muenster, Germany; y.rellmann@uni-muenster.de (Y.R.); eidhof@uni-muenster.de (E.E.); 2 Institute of Musculoskeletal Medicine, University Hospital Münster, Albert-Schweitzer-Campus 1, Building D3, 48149 Muenster, Germany; uhansen@uni-muenster.de; 3 Center for Applied Tissue Engineering and Regenerative Medicine-CANTER, Munich University of Applied Sciences, 80335 Munich, Germany; lutz.fleischhauer@hm.edu (L.F.); jvogel@hm.edu (J.V.); hauke.clausen-schaumann@hm.edu (H.C.-S.); 4 Center for Nanoscience-CeNS, 80335 Munich, Germany; 5 Department for Orthopaedics and Trauma Surgery, Musculoskeletal University Center Munich (MUM), University Hospital, LMU Munich, 80335 Munich, Germany; attila.aszodi@med.uni-muenchen.de

**Keywords:** cartilage, ER stress, osteoarthritis, apoptosis, osteophytes

## Abstract

Ageing or obesity are risk factors for protein aggregation in the endoplasmic reticulum (ER) of chondrocytes. This condition is called ER stress and leads to induction of the unfolded protein response (UPR), which, depending on the stress level, restores normal cell function or initiates apoptotic cell death. Here the role of ER stress in knee osteoarthritis (OA) was evaluated. It was first tested in vitro and in vivo whether a knockout (KO) of the protein disulfide isomerase ERp57 in chondrocytes induces sufficient ER stress for such analyses. ER stress in ERp57 KO chondrocytes was confirmed by immunofluorescence, immunohistochemistry, and transmission electron microscopy. Knee joints of wildtype (WT) and cartilage-specific ERp57 KO mice (ERp57 cKO) were analyzed by indentation-type atomic force microscopy (IT-AFM), toluidine blue, and immunofluorescence/-histochemical staining. Apoptotic cell death was investigated by a TUNEL assay. Additionally, OA was induced via forced exercise on a treadmill. ER stress in chondrocytes resulted in a reduced compressive stiffness of knee cartilage. With ER stress, 18-month-old mice developed osteoarthritic cartilage degeneration with osteophyte formation in knee joints. These degenerative changes were preceded by apoptotic death in articular chondrocytes. Young mice were not susceptible to OA, even when subjected to forced exercise. This study demonstrates that ER stress induces the development of age-related knee osteoarthritis owing to a decreased protective function of the UPR in chondrocytes with increasing age, while apoptosis increases. Therefore, inhibition of ER stress appears to be an attractive therapeutic target for OA.

## 1. Introduction

Osteoarthritis (OA) is the most prevalent musculoskeletal condition, currently affecting an estimated 250 million people worldwide. The degenerative joint disease is characterized by a progressive loss of joint cartilage, synovial inflammation, formation of osteophytes, and subchondral bone remodeling. Despite a substantial amount of research, the multifactorial etiology and the complex pathogenesis of OA are not yet entirely understood. However, age-related changes in articular cartilage homeostasis, causing an imbalance between the synthesis and breakdown of essential components of the extracellular matrix (ECM), are definitely crucially involved [1,2,3,4].

Under physiological conditions, articular chondrocytes maintain the constituents of their surrounding ECM in a low-turnover state of equilibrium [5]. Under pathological conditions, chondrocytes can increase the production of ECM components, e.g., as a first reaction to cartilage damage [6]. All proteins, glycoproteins, and proteoglycans, destined for the ECM, are synthesized at the rough endoplasmic reticulum (ER). During translation, the polypeptide chains are translocated into the ER lumen, where they are post-translationally modified and subsequently folded with the help of ER resident molecular chaperones. Only correctly folded proteins move via vesicular transport to the Golgi apparatus, where additional modifications occur and sorting into vesicles is established. This enables further transport to different cellular compartments or secretion into the extracellular space [7].

In the case of incorrect folding, proteins accumulate in the ER cisternae [8,9]. This condition is referred to as ER stress and causes ER dysfunction. To counteract ER stress and restore the functions of the ER, the unfolded protein response (UPR) is initiated [10]. The UPR has different effects depending on the level of damage caused by the ER stress. If the ER stress is low, translation processes are stopped and signaling pathways are activated, which leads to an increased synthesis of additional folding proteins (e.g., Binding immunoglobulin Protein (BiP), calnexin, or calreticulin). Moreover, the ER-associated degradation (ERAD), i.e., the breakdown of misfolded proteins in proteasomes after ubiquitination or by autophagy, is initiated. If the ER stress is high or persistent, the UPR leads to apoptotic chondrocyte death. In growth plate cartilage, the UPR also induces changes in differentiation and cell-cycle progression of hypertrophic chondrocytes [11]. Because hypertrophic differentiation and apoptotic cell death are hallmarks of OA [12,13], a proper ER function in chondrocytes is not only essential for effective protein synthesis and secretion into the ECM, but could also prevent cartilage degeneration [7].

ERp57 (or PDIA3) is a member of the protein disulfide isomerase (PDI) family of ER resident chaperones and is involved in disulfide bridge formation [14]. It acts in complex with calnexin and calreticulin, both of which recognize specific sugar residues on unfolded polypeptide chains. Consequently, ERp57 almost exclusively catalyzes disulfide bond formation in glycoproteins [15]. Most ERp57 substrates are heavily glycosylated disulfide-bonded proteins that share common structural domains. These include various ECM proteins, such as collagen IV, laminins, and agrin, or ECM receptors such as integrins [16,17]. By elimination of disarranged disulfide bridges and the formation of new ones, ERp57 also accounts for a folding correction [18].

ERp57-dependent PDI activity in chondrocytes was shown to be essential for postnatal skeletal growth in mice, especially during the pubertal growth spurt characterized by intensive protein synthesis. ERp57 deficiency resulted in an obvious chondrodysplasia-like bone phenotype with protein aggregation in the ER, initiation of the UPR, reduced proliferation, and accelerated apoptotic cell death of growth plate chondrocytes [19]. Since some features of endochondral ossification are similar to those in degenerative cartilage diseases [20], this study focuses on the role of ER stress in the development of osteoarthritis.

## 2. Results

### 2.1. ERp57 Deficiency in Chondrocytes Leads to ER Stress In Vitro and In Vivo

**Figure 1:**
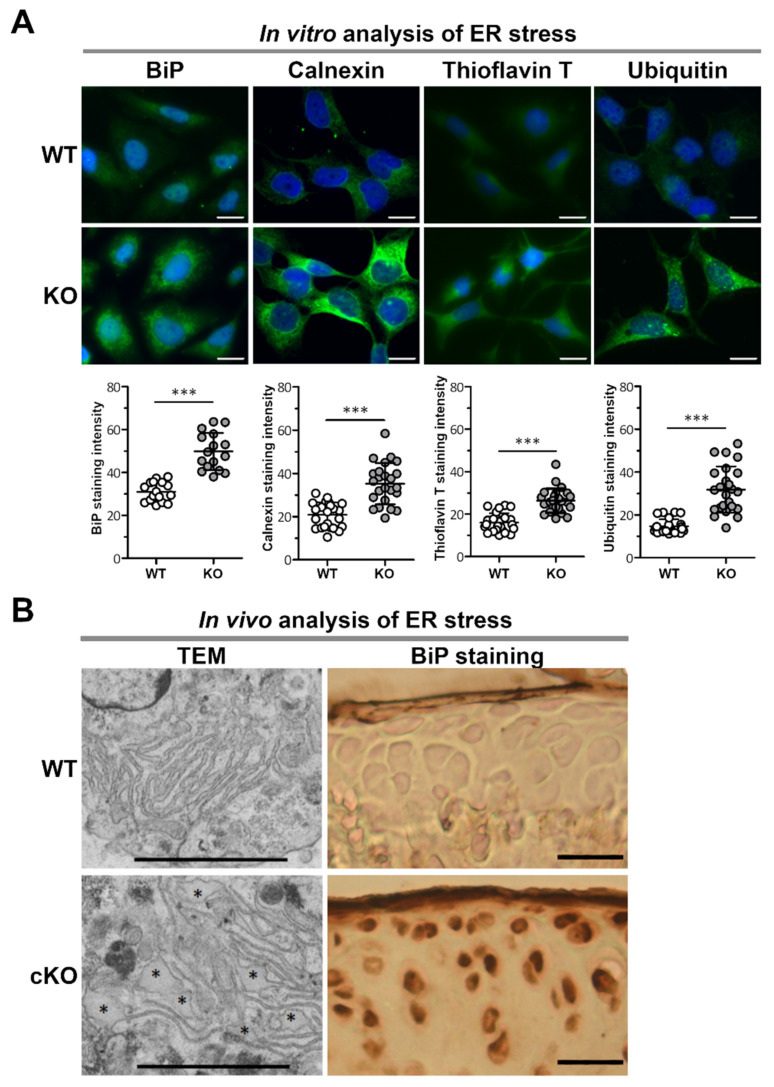
Lack of the protein disulfide isomerase ERp57 in chondrocytes results in ER stress in vitro and in vivo. (**A**) In vitro analysis of CRISPR/Cas9-generated C28/I2 ERp57 KO cells by immunofluorescence staining shows increased levels of the ER stress marker proteins BiP (*p* = 0.000000014) and calnexin (*p* = 0.000000087) compared to WT control cells. In the endoplasmic reticulum, the formation of protein aggregates detected by thioflavin T staining (*p* = 0.000000009) and the ER-associated degradation of proteins indicated by the presence of ubiquitinylated proteins (*p* = 0.000000001) are correspondingly increased in KO cells compared to WT controls (*n* = 3). Scale bars = 20 µm. (**B**) In vivo analysis of isolated chondrocytes from knee and hip cartilage of newborn WT and ERp57 cKO mice by transmission electron microscopy (TEM) shows a normal ER structure in WT cells, while dilated ER cisternae (*) are detectable in ERp57 cKO chondrocytes (*n* = 3). Immunohistochemical staining of articular knee cartilage cryosections from 14-week-old mice revealed higher staining intensity of the ER stress marker protein BiP in the ERp57 cKO animals compared to WT mice (*n* = 3). Scale bars TEM = 1 µm, scale bars BiP staining = 50 µm. *** = *p* < 0.005.

Immunofluorescence analysis of C28/I2 KO cells revealed that the ER stress markers BiP and Calnexin were expressed at increased levels compared to C28/I2 WT cells (Figure 1A). Both markers are chaperone proteins and part of the folding machinery in the ER, which is upregulated in the context of the UPR initiated by ER stress. Furthermore, many more protein aggregates that form due to misfolding events were detected in KO cells by Thioflavin T (ThT) compared to WT cells (Figure 1A). Upon binding to hydrophobic stretches in protein aggregates, ThT displays excitation and emission of fluorescence. Elevated ThT fluorescence directly correlates with increased activation of the UPR [21]. The cells were also stained for ubiquitin (Figure 1A), which is a marker for ER-associated degradation (ERAD). In this process, misfolded and/or aggregated proteins are ubiquitinated and thereby submitted to proteasomal degradation. In C28/I2 KO cells, higher amounts of ubiquitin were observed, indicating increased degradation of protein aggregates via ERAD.

To demonstrate that ER stress is also present in the cartilage of ERp57 cKO mice, chondrocytes derived from the knee cartilage of WT and ERp57 cKO mice were analyzed by TEM (Figure 1B left). An elevated amount of dilated ER cisternae as a sign of protein aggregation in the ER was detected in ERp57 cKO cartilage. In contrast, the ER of WT cartilage appeared to be well structured. In addition, cryosections of the knee cartilage of 14-week-old WT and ERp57 cKO mice were used to analyze the amount of the ER stress marker protein BiP. BiP staining was increased in the articular cartilage of ERp57 cKO mice (Figure 1B right).

Collectively, these results confirm that the loss of ERp57 in both C28/I2 cells in vitro and knee cartilage of the mouse model in vivo induces ER stress through protein aggregation in the ER. Consequently, the ERp57 cKO mouse is a suitable model for analyzing the role of ER stress in osteoarthritis.

### 2.2. ER Stress in Chondrocytes Results in Reduced Compressive Stiffness of Knee Cartilage

**Figure 2:**
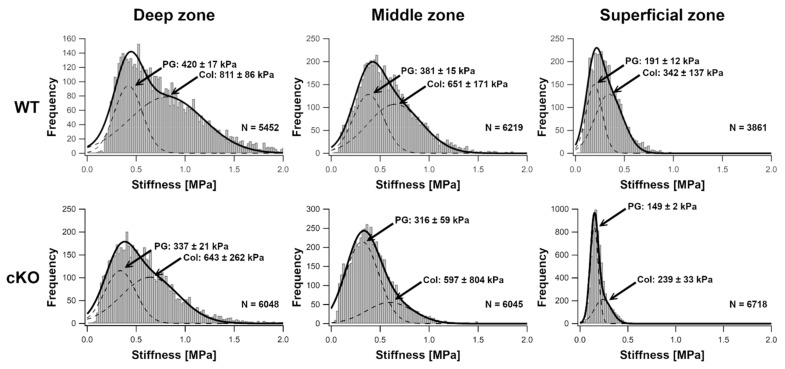
ERp57 cKO mice display reduced compressive stiffness of knee cartilage. Histograms of Young’s moduli obtained by indentation-type atomic force microscopy (IT-AFM) reveal bimodal Young’s modulus distributions. The low modulus peak depicts the proteoglycan moiety, whereas the second peak represents the collagen fibril network (indicated by arrows, PG = proteoglycan, Col = collagen). All values are depicted as peak values ± 2-fold standard deviation of the fitted curve. This corresponds to a confidence interval of 95%. In 14-week-old ERp57 cKO animals, the shifts of the proteoglycan and collagen peaks towards lower values compared to WT mice demonstrates a decreased compressive stiffness in all three zones of the knee joint cartilage under ER stress. N = Number of individual data points.

Indentation-type atomic force microscopy (IT-AFM) was applied to native tissue sections of knee joint cartilage from 14-week-old WT and ERp57 cKO mice to analyze compressive stiffness in the presence and absence of ER stress at the nanoscale. In both WT and ERp57 cKO mice, histograms with bimodal stiffness distributions were observed, with the lower stiffness peak attributable to the proteoglycan moiety and the higher stiffness peak associated with the collagen fibrils [22,23,24]. As shown in Figure 2 and Supplementary Figure S3, stiffness peaks for the proteoglycan network were observed in ERp57 cKO cartilage at 337 ± 21 kPa in the deep zone, 316 ± 59 kPa in the middle zone, and 149 ± 2 kPa in the superficial zone. In WT cartilage, the average proteoglycan stiffness values were higher in all zones: 420 ± 17 kPa in the deep zone, 381 ± 15 kPa in the middle zone, and 191 ± 12 kPa in the superficial zone. In ERp57 cKO samples, the stiffness peaks of collagen fibrils were 643 ± 262 kPa in the deep zone, 597 ± 804 kPa in the middle zone, and 239 ± 33 kPa in the superficial zone; whereas in the WT cartilage, the average stiffness of collagen fibrils was higher: 811 ± 86 kPa in the deep zone, 651 ± 171 kPa in the middle zone, and 342 ± 137 kPa in the superficial zone.

In summary, both components of the cartilage ECM, the proteoglycans and the collagens, displayed a reduced stiffness in ERp57 cKO knee joints. This indicates that ER stress in knee cartilage compromises the biomechanical properties of the extracellular matrix, making this tissue potentially more susceptible to cartilage degeneration processes such as osteoarthritis.

### 2.3. Aged ERp57 cKO Mice Show Signs of Osteoarthritis

**Figure 3:**
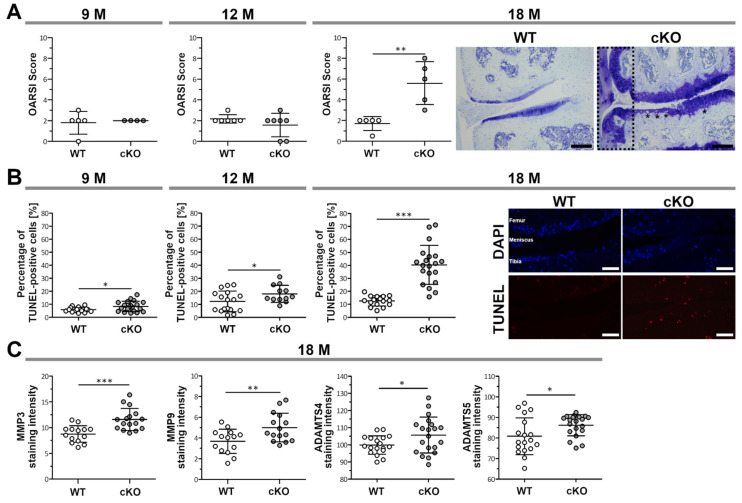
ERp57 cKO mice show an accelerated development of osteoarthritis during aging. (**A**) Histological analysis and OARSI scoring of toluidine blue-stained knee sections of 18-month-old mice reveals an increase in cartilage degradation, the occurrence of hypertrophic chondrocytes (*), and osteophyte formation (scattered box) in ERp57 cKO mice, but not in WT animals (*p* = 0.0039). No significant difference was detected between younger WT and ERp57 cKO mice at the age of 9 months (*p* = 0.7924) and 12 months (*p* = 0.2503). Scale Bars = 100 µm. (**B**) TUNEL staining indicates a higher percentage of apoptotic cells in the articular cartilage of ERp57 cKO mice at 9 (*p* = 0.0271), 12 (*p* = 0.0490) and 18 months (*p* = 0,000000028) compared to WT animals. Scale bars = 50 µm (**C**) Immunofluorescence of 18-month-old ERp57 cKO cartilage reveals increased staining intensities of matrix degrading proteases MMP3 (*p* = 0.0004), MMP9 (*p* = 0.0097), ADAMTS4 (*p* = 0.0428), and ADAMTS5 (*p* = 0.0288) compared to WT controls. M = months. n ≥ 4. *** *p* < 0.005, ** *p* < 0.01, * *p* < 0.05.

To monitor the spontaneous development of OA during ageing, knee articular cartilage of WT and ERp57 cKO mice was subjected to histological analysis at 9, 12, and 18 months of age. Toluidine blue O-stained knee joints were evaluated using a modified OARSI scoring to determine cartilage degradation and osteophyte formation (Figure 3A and Supplementary Figure S1). No significant differences were observed between OARSI scores of 9- and 12-month-old WT and ERp57 cKO mice. However, at the age of 18 months, cartilage degradation was significantly increased in ERp57 cKO animals. In the most severe cases, the knee joint in ERp57 cKO animals showed erosion of the cartilage down to the calcified zone at around 50–75% of the articular surface area, especially at the medial tibial plateau of the joint. In addition to cartilage degradation, osteophyte formation (Figure 3A, scattered box and Supplementary Figure S1) was observed in ERp57 cKO knee samples, which is likewise a common feature of osteoarthritis [25]. Furthermore, larger hypertrophic chondrocytes (see also Supplementary Figure S1), which were collagen X positive (data not shown), were detectable in severely degenerated areas of the ERp57 cKO cartilage but not in WT tissue.

TUNEL staining has revealed that increased apoptosis of articular chondrocytes in cKO mice precedes cartilage degeneration (Figure 3B). Throughout all analyzed timepoints, approximately 10% of the chondrocytes in WT cartilage were apoptotic. In ERp57 cKO animals, the apoptotic rate starts to increase at 9 months of age, and by 18 months, when cartilage degradation is also enhanced, the percentage of apoptotic chondrocytes increases significantly to about 40%.

Matrix metalloproteinases (MMPs) are capable of degrading a variety of ECM components, such as collagens, fibronectin, laminin, elastin, and several proteoglycans, and are described to be upregulated in osteoarthritic cartilage [26,27,28]. Two enzymes, the disintegrin and metalloproteinases with thrombospondin motifs (ADAMTS) -4 and -5, also referred to as aggrecanases, degrade aggrecan [29,30]. Quantification of staining intensities revealed that the expression of MMP3, MMP-9, ADAMTS4, and ADAMTS5 were significantly increased in 18-month-old ERp57 cKO articular cartilage (Figure 3C); see also Supplementary Figure S2.

Thus, the accelerated cartilage degradation found in aged ERp57 cKO mice could be the consequence of the reduced compressive stiffness, increased number of apoptotic chondrocytes, or higher amounts of ECM-degrading MMPs and aggrecanases in the articular cartilage. It is most likely that a combination of all the changes described results in faster cartilage degradation.

### 2.4. Young ERp57 cKO Mice Are Not Susceptible to Osteoarthritic Cartilage Degeneration, Even When Subjected to Forced Exercise

**Figure 4:**
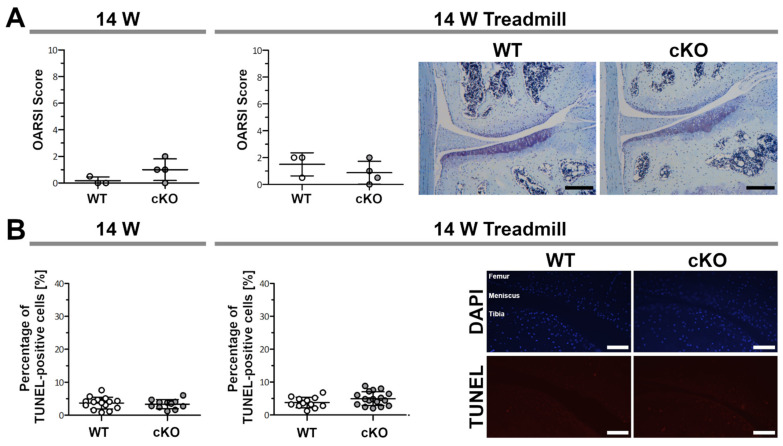
Young ERp57 cKO mice are not susceptible to osteoarthritic cartilage degeneration even after forced treadmill running. (**A**) At the age of 14 weeks, WT and ERp57cKO mice showed no difference in OARSI scoring on toluidine blue-stained sections (*p* = 0.1583). Same-aged mice that were forced to run on a treadmill for 40 min/day for 6 weeks (14 W Treadmill) exhibit similar OARSI scores (*p* = 0.3844). Scale bars = 100 µm. (**B**) TUNEL staining to detect apoptotic chondrocytes showed no difference between WT and ERp57 cKO mice after treadmill exercise (*p* = 0.1179) and in the age-matched control group (*p* = 0.5949). Scale bars = 50 µM. W = weeks. *n* ≥ 3.

To examine whether cartilage degeneration in young animals could be induced by overload through forced exercise, 8-week-old WT and ERp57 cKO mice were treadmilled for 40 min a day, 5 days a week for 6 weeks. Osteoarthritic changes in the treadmill group were then analyzed in comparison to a control group of the same age (14 weeks) without exercise. OARSI scoring revealed no differences between WT and ERp57 cKO animals in either the control group or the treadmill group (Figure 4A). The articular cartilage appeared healthy in all of the mice examined and had a smooth surface without fissures or clefts. The mean OARSI score was below 2 in all animals examined.

Furthermore, TUNEL assays on these knee sections showed a very low number of apoptotic chondrocytes and no difference in the percentages of apoptotic cells between WT and ERp57 cKO animals, neither in the control nor in the treadmill group (Figure 4B).

In summary, these observations suggest that treadmill running is insufficient to induce apoptotic cell death and osteoarthritic cartilage degeneration in the knee joints of young mice with ER-stressed cartilage.

## 3. Discussion

In the present study, IT-AFM analysis demonstrated that the loss of ERp57 in chondrocytes results in reduced compressive stiffness of the articular cartilage. This is considered to be a prerequisite for promoting cartilage degeneration processes or to represent already early pathophysiological changes in the development of OA [31]. The changes in articular cartilage stiffness in the ERp57 cKO mice could be a consequence of impaired ER folding capacity, which results in a reduction in the total amount of functional ECM components. On the other hand, the cross-linking of proteoglycans or collagens within the ECM could be disturbed by missing glycoproteins, which are normally folded by the calnexin-calreticulin-ERp57-complex. However, these biomechanical changes alone are most likely not sufficient to trigger late-stage OA. Severe cartilage degradation and osteophyte formation are likely to occur only in conjunction with additional aberrations such as ER stress-induced changes in cartilage homeostasis.

ERp57 deficiency in chondrocytes resulted in the accumulation of protein aggregates in ER cisternae and induced the expression of ER stress marker proteins such as BiP. This appears to be the case in all types of chondrocytes, as observed here in knee articular cartilage and previously in growth plate cartilage [19]. The ERp57 cKO mouse was used here as a model system to analyze the role of ER stress in knee joint diseases such as OA.

A growing number of mouse models have been developed in which protein folding processes in chondrocytes are changed. Most of them were used to analyze the role of ER stress in chondrodysplasias such as metaphyseal chondrodysplasia type Schmid (MCDS), multiple epiphyseal dysplasia (MED), or pseudoachondrodysplasia (PSACH) [7,32]. In the meantime, however, some ER stress models have also been investigated with regard to osteoarthritis [33]. The most recent development is the I-ERS mouse, in which ER stress is generated by the expression of mutant cartilage oligomeric matrix protein (COMP) as a misfolded protein in adult articular chondrocytes to simulate non-trauma-induced, age-dependent OA. In this mouse model, ER stress-induced osteoarthritic damage occurred slowly, closely mimicking cartilage degeneration in the elderly [34].

**Figure 5:**
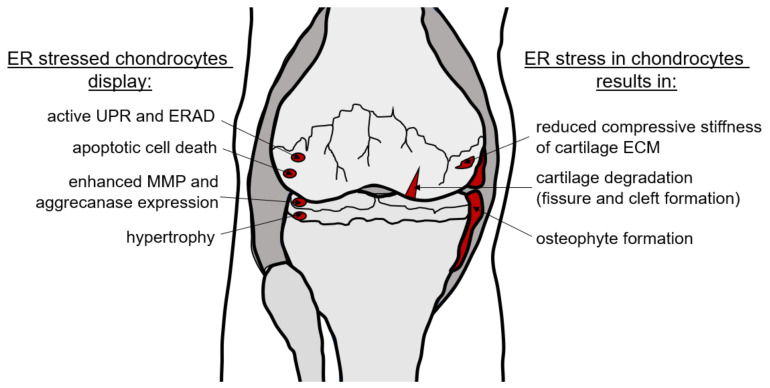
ER stress-induced osteoarthritic changes in the knee joint. Knee joints of aged ERp57 cKO animals display severe OA cartilage degeneration with enhanced MMP3, MMP9, ADAMTS4, and ADAMTS5 expression and osteophyte formation. These changes are preceded by reduced compressive stiffness of the cartilage ECM and increased apoptotic cell death. Both effects are likely induced by ER stress that cannot be adequately compensated by the UPR.

As OA involves numerous risk factors and various pathogenetic factors, different ER stress models are required to assess the complexity of this joint disease. The ERp57 cKO mouse was used here to analyze age-dependent and overuse-induced OA triggered by ER stress. ERp57 does not appear as an OA-relevant gene in disease databases because loss-of-function mutations in all cells lead to embryonic lethality [35]. Therefore, ERp57 mutations themselves are most likely irrelevant for OA development. However, in cartilage-specific KO mice, severe ER stress is induced in chondrocytes without loss of function of a specific ECM protein, making the ERp57 cKO mouse an excellent model for the analysis of ER stress in vivo. All the changes observed in the knee joint, such as the degradation of articular cartilage with increased MMP and aggrecanase expression, chondrocyte hypertrophy and apoptosis, reduced compressive stiffness of collagens and proteoglycans in the cartilage ECM, and osteophyte formation are schematically summarized in Figure 5.

In general, ER stress activates the UPR as a rescue mechanism to restore normal cell function. This defense mechanism works very well in the articular cartilage of young ERp57 cKO mice, and even after exercise-induced overload the joint cartilage remains healthy and functionally intact. In contrast, the UPR is less effective in aged animals. Normal cartilage function cannot be maintained in aged animals and apoptotic cell death is induced in cartilage cells.

Ageing is a risk factor for OA [36] and the age-related lack of ER stress compensation is likely to contribute to cartilage tissue damage. As has been described, chondrocytes from aged articular cartilage display decreased expression of ER stress-induced chaperones such as PDI, calnexin, and Ero1-like protein alpha. In addition, increased immunohistochemical staining was detected for several ER stress markers such as phosphorylated IRE1 alpha, spliced X-box binding protein 1 (XBP1), activating transcription factor-4 (ATF-4), and C/EBP homologous protein (CHOP), as well as for apoptotic markers such as cleaved caspase 3 and cleaved poly(ADP-ribose) polymerase, suggesting that the decrease in chaperones during ageing induces ER stress and chondrocyte apoptosis [37]. A shift in the balance between the protective, adaptive response of the UPR and pro-apoptotic signaling during ageing has been described in various tissues, with a significant decrease in the protective arm and a robust increase in the apoptotic arm [38]. This could be due to the fact that ER chaperones show oxidative damage with increasing age, which limits their function in protein homeostasis [39].

The accumulation of advanced glycation end products (AGEs) on nucleotides, lipids, and peptides/proteins is an inevitable part of the ageing process in all organisms, including humans [40,41]. AGEs can induce or enhance ER stress in two ways. Intracellularly, AGEs colocalize with chaperones such as BiP to form high molecular weight complexes [42], leading to their dysfunction [43]. Extracellularly formed AGEs induce ER stress by binding to receptors of AGEs (RAGEs), which are found on chondrocytes to be increasingly expressed with age. This signaling has been described to contribute to the development and/or progression of OA [42,44,45,46]. Of note, in line with our observations on ER stress-induced OA, young RAGE KO mice were shown to be protected from developing OA after knee destabilization surgery [47]. In summary, aged cartilage is more susceptible to chondrocyte apoptosis and cartilage degeneration, as ER stress occurs more frequently in old age, due to the decreased expression or functionality of ER chaperones or the accumulation of ER stress-inducing AGEs.

Another risk factor for OA is obesity. However, in mice, Tan et al. observed that it is not overweight itself, but a dietary overload of the saturated fatty acid palmitate that promotes the occurrence of cartilage lesions in knee joints. These lesions were shown to develop because palmitate-induced ER stress triggered the UPR and enhanced apoptosis in chondrocytes [48]. Therefore, diet-related ER stress also plays a crucial role in OA initiation, as in other obesity-related disorders [49].

The fact that ER stress plays a crucial role in the development of age-related or obesity-induced osteoarthritis opens up new options for the therapeutic management of osteoarthritis. In addition to a healthy diet to prevent diet-related OA development, agents that reduce ER stress or reduce apoptotic events in chondrocytes hold promise. This includes (1) small molecular chemical chaperones such as 4-PBA, which efficiently support protein folding in the ER and ameliorate UPR signaling [50]; (2) inhibitors of single UPR pathways, such as ISRIB, that inhibits eIF2α phosphorylation on the PERK signaling arm to reduce apoptosis [51]; (3) activators of autophagy and ER-associated degradation, such as carbamazepine [52]; as well as (4) inhibitors of excessive apoptosis. However, how these or other adequate compounds could be used in the future and whether combinatorial therapy might be even more beneficial to achieve an improvement in ER stress-related cartilage degeneration in patients remains to be investigated in future studies.

## 4. Materials and Methods

### 4.1. Cartilage-Specific ERp57 Knockout Mice (ERp57 cKO)

ERp57 cKO mice were generated as described [19]. Homozygous ERp57 floxed Col2a1-cre-positive mice (ERp57^fl/fl^-Col2a1-cre) were mated with homozygous ERp57 floxed mice (ERp57^fl/fl^) to gain WT (ERp57^fl/fl^) and ERp57 cKO (ERp57^fl/fl^-Col2a1-cre) littermates. To exclude gender-specific differences, all analyses were performed with male mice only. To analyze OA development, young mice (14-week-old animals) with or without treadmill exercise and aging mice without an additional application of stress (9, 12, 18-month-old animals) were used. The mice were kept under pathogen-free conditions and supplied with food and water ad libitum in a 12-h light/dark cycle in compliance with the German federal law for animal protection under the control of the North Rhine-Westphalia State Agency for Nature, Environment, and Consumer Protection (LANUV, NRW, AZ 84-02.04.2017.A192).

### 4.2. Culture of C28/I2 WT Cells and ERp57 Knockout C28/I2 Cells

C28/I2 WT chondrocytes [53] were cultured at 37 °C, 5% CO_2_, and 100% humidity in DMEM (Biochrom, Berlin, Germany) supplemented with 10% FCS, 1% sodium pyruvate, 100 units/mL penicillin, and 100 μg/mL streptomycin (DMEM complete). ERp57 knockout C28/I2 cells (C28/I2 KO) were generated using CRISPR/Cas9 as previously described [50] and cultured likewise.

### 4.3. (Immuno-) Fluorescence Analysis of C28/I2 Cells

C28/I2 WT and KO cells (15.000 per well) were seeded in DMEM complete on 8-well IBIDI slides with a removable chamber (80841, ibidi GmbH, Gräfelfing, Germany). After 3 h, the medium was changed to FCS-free DMEM complete and supplemented with 60 μg/mL β-aminopropionitrile fumarate, 25 μg/mL sodium ascorbate, 1 mM cysteine, and 1 mM pyruvate (ABCP). The cells were cultured in this medium for 24 h at 37 °C, 5% CO_2_, and 100% humidity. For immunofluorescence staining, cells were fixed with 1% paraformaldehyde in PBS at pH 7.4 (PFA/PBS) for 10 min at room temperature (RT), washed twice for 5 min with PBS, and then incubated in ethanol:acetic acid (2:1) for 5 min at −20 °C. After washing with PBS (3 times for 5 min), unspecific protein binding was blocked for 1 h with 5% BSA in PBS at RT, and the samples were subsequently incubated overnight at 4 °C with the primary antibody diluted in 2% BSA/PBS. To analyze ER stress, polyclonal antibodies against BiP (ADI-SPA-768-0050, Enzo Life Science, Farmindale, NY, USA, 1:100) and Calnexin (ADI-SPA-860D, Enzo Life Science, USA, 1:100) were used. To analyze ubiquitinylated proteins destined for ER-associated degradation, the anti-ubiquitin antibody (ab134953, Abcam, Cambridge, UK, 1:250) was applied. The samples were rinsed with PBS (3 times for 5 min) and then incubated with Alexa Fluor^®^ 488-labelled donkey anti-rabbit secondary antibody (A-21206, Invitrogen, West Grove, PA, USA,1:500) in 2% BSA/PBS for 1 h at RT in the dark. After three washing steps with PBS for 5 min, the silicone chambers of the IBIDI slides were removed and the slides were mounted with Fluoromount + DAPI (00-4959-52, Thermo Fisher, Waltham, MA, USA). To stain protein aggregates 24 h after seeding, the cells were incubated overnight with 5 µM Thioflavin T at 37 °C. Subsequently, the samples were rinsed with PBS (3 times for 5 min) and fixed with 4% PFA/PBS. After further washing steps with PBS (3 × 5 min), the silicone chambers of the IBIDI slides were removed and the slides were mounted with Fluoromount + DAPI (00-4959-52, Thermo Fisher, Waltham, MA, USA). Images were taken using a Leica DMi8 automated microscope (Leica Microsystems, Wetzlar, Germany).

### 4.4. Transmission Electron Microscopic Analysis (TEM)

Knee cartilage was dissected from newborn WT and ERp57 cKO mice and incubated overnight in DMEM supplemented with 1 mg/mL Collagenase B (Roche, Mannheim, Germany) and 1 mM cysteine. Next, 400,000 of these isolated cells were pipetted as 20 µL droplets of cells in DMEM to form micromass cultures in a 24-well plate. After 3 h, 1 mL DMEM complete supplemented with ABCP was added to the micromass cultures, which were then cultivated for 7 days at 37 °C and 5% CO_2_. Micromasses were then fixed overnight with 2% (*v*/*v*) PFA and 2.5% (*v*/*v*) glutaraldehyde in 100 mM cacodylate buffer pH 7.4 at 4 °C. Micromasses were washed in PBS and postfixed in 0.5% (*v*/*v*) osmium tetroxide and 1% (*w*/*v*) potassium hexacyanoferrate (III) in 0.1 M cacodylate buffer at 4 °C for 2 h. After another washing step with distilled water, the samples were dehydrated in an ascending ethanol series and subsequently incubated in propylene oxide (2 × 15 min). Micromasses were embedded in Epon and ultrathin sections were cut with an ultramicrotome. Sections were collected on copper grids and negatively stained with 2% uranyl acetate for 10 min. A Phillips EM-410 electron microscope was used to take electron micrographs at 60 kV using imaging plates (Ditabis, Pforzheim, Germany).

### 4.5. Indentation-Type Atomic Force Microscopy (IT-AFM)

Knees from 14-week-old WT and ERp57 cKO mice were dissected, embedded in O.C.T. compound mounting medium (00411243, VWR chemicals, Darmstadt, Germany), and frozen in liquid nitrogen. A Leica CM1950 cryostat (Leica Biosystems, Wetzlar, Germany) was used to generate 20 µm-thick frozen tissue sections. Sections were thawed, submerged in PBS buffer, and analyzed with a NanoWizard I AFM (JPK Instruments, Berlin, Germany) in combination with an inverse optical microscope (Axiovert 200, Zeiss, Göttingen, Germany) as described before [23,54,55]. Briefly, silicon nitride cantilevers (0.1 N/m, MLCT, Bruker) were used to record 625 indentation curves in an area of 3 × 3 µm^2^. For each sample, 9 areas were measured. The sample stiffness was determined by fitting a modified Hertz model to the force-indentation curves using the JPK Data Processing software (V5.0.96, JPK Instruments, Berlin, Germany). Histograms of the Youngs Moduli were generated and a linear combination of two Gaussian functions was fitted to the histograms utilizing Igor Pro software (Version 6.3.7.2, WaveMetrics).

### 4.6. Treadmill Experiments

Forced exercise on a treadmill [56] was used to induce osteoarthritic changes in the knee cartilage of WT and ERp57 cKO mice. Eight-week-old mice ran on a motorized treadmill with a 20% incline for 40 min/day, 5 times a week, and 6 weeks in total. The running speed was set to 0.28 m/s ± 0.06 m/s, which refers to 80% maximum oxygen consumption. After exercise, mice were sacrificed, and knee samples were embedded in paraffin.

### 4.7. Sample Preparation for Paraffin- and Cryosections of Cartilage Tissues

Hind legs of mice at the age of 14 weeks (young mice with or without forced exercise on a treadmill) as well as 9 months, 12 months, and 18 months (ageing mice without additional application of stress) were dissected. For paraffin-embedding, legs were fixed with 4% PFA in PBS at 4 °C overnight and decalcified in 20% ethylene diamine tetraacetic acid (EDTA) with 6.6% (*w*/*v*) Tris under rotation for 2 weeks at RT. During this time, the decalcifying solution was changed every 2 days. Subsequently, legs were dehydrated by a graded series of ethanol and isopropanol solutions and embedded in paraffin (Paraplast, X880.1, Roth, Germany). Moreover, 4.5-µm-thick sections were cut through the frontal plane of the knee joint with a rotation microtome AM355 (Microm, Wetzlar, Germany) and collected on glass slides. For the preparation of cryosections, dissected unfixed hind legs were embedded in O.C.T. compound mounting medium (00411243, VWR chemicals, Darmstadt, Germany), frozen in liquid nitrogen, and cut through the sagittal plane of the knee joint with a Leica CM1950 cryostat (Leica Biosystems, Wetzlar, Germany). Lastly, 4.5-µm-thick cryosections were collected on crystal clear book repair adhesive tape [57].

### 4.8. Histological Staining of Cartilage Samples

Paraffin-embedded knee sections were deparaffinized, stained with 0.2% (*w*/*v*) Toluidine blue O (Serva, Germany) in 0.2 M sodium acetate buffer pH 4.2 for 10 min at RT, and mounted with Entellan (Merck, Gernsheim, Germany). Images of the knee joints were taken with a Nikon SMZ25 stereo microscope (Nikon Instruments, Tokyo, Japan).

### 4.9. Osteoarthritis Scoring

Toluidine blue O-stained frontal knee joints were analyzed by OARSI scoring [58]. The scores include OA-linked cartilage degradation with a grading from 0 to 6 and osteophyte formation together with changes of the subchondral bone with a grading from 0 to 3, which were summed up to a maximal score of 9. Scores were obtained for medial femoral condyle, medial tibial plateau, as well as lateral femoral condyle and lateral tibial plateau. The OA severity is expressed as a maximal score, based on all four quadrants.

### 4.10. Immunohistochemical and Immunofluorescence Analysis of Cartilage Samples

To evaluate the protein expression of BiP in articular knee cartilage, cryosections were fixed in 1% PFA/PBS for 10 min at RT. The sections were rinsed with PBS and then incubated with Chondroitinase ABC (0.2 U/mL) in 60 mM sodium acetate, 0.02% (*w*/*v*) BSA and 50 mM Tris, pH 8.0 for 90 min at 37 °C. After washing with PBS, endogenous peroxidase activity was blocked with 3% (*v*/*v*) H_2_O_2_ for 10 min at RT. The sections were washed again in PBS. BiP staining was carried out using the Vectastain^®^ Elite ABC Universal Kit (VEC-PK-7200, BIOZOL, Eching, Germany) and the Vector^®^ DAB Peroxidase Substrate (VEC-SK-4100, BIOZOL, Eching, Germany) with an anti-BiP polyclonal antibody (ADI-SPA-768-0050, Enzo Life Science, Farmingdale, NY, USA, 1:200) according to the manufacturer’s instructions. Sections were dried at RT and mounted with Entellan. To assess the expression of MMP3, MMP9, ADAMTS4, and ADAMTS5, paraffin-embedded knee sections were deparaffinized, rehydrated, and then preincubated in 0.05% (*w*/*v*) protease XXIV in PBS for 10 min at 37 °C and 0.1% (*w*/*v*) hyaluronidase in acetate buffer pH 6.0 for 90 min at 37 °C. Sections were rinsed in PBS. For MMP3 and MMP9 staining, sections were incubated with 5% (*w*/*v*) BSA/PBS for 60 min at RT to block unspecific protein binding. The incubation with the anti-MMP3 antibody (MAB3321, Merck, Gernsheim, Germany, 1:100) or the anti-MMP9 antibody (ab38898, abcam, Cambridge, UK, 1:200) was carried out in 2% (*w*/*v*) BSA/PBS at 4 °C overnight. As the secondary antibody, the Alexa Fluor^®^ 488 donkey anti-rabbit antibody (1:500, A-21206, Invitrogen, Waltham, MA, USA) for MMP9 staining and the Alexa Fluor^®^ 568 donkey anti-rabbit antibody (1:500, A-10042, Invitrogen, Waltham, MA, USA) for MMP3 staining were used. Both antibodies were diluted in 2% (*w*/*v*) BSA/PBS and incubated for 1 h at RT. The sections were rinsed in PBS and mounted with Fluoromount + DAPI (00-4959-52, Thermo Fisher, Waltham, MA, USA). For ADAMTS4 and ADAMTS5 staining, endogenous peroxidase activity was blocked with 3% (*v*/*v*) H_2_O_2_ for 10 min at RT. Then the Vectastain^®^ Elite ABC Universal Kit (VEC-PK-7200, BIOZOL, Eching, Germany) and the Vector^®^ DAB Peroxidase Substrate (VEC-SK-4100, BIOZOL, Eching, Germany) were used according to the manufacturer’s instructions with polyclonal antibodies (anti-ADAMTS4: bs-4191R, Bioss Antibodies Inc., Woburn, MA, USA, 1:200 and ADAMTS5: bs-3573R, Bioss Antibodies Inc, Woburn, MA, USA, 1:50), which were incubated on the slides overnight at 4 °C. Sections were dried at RT and mounted with Entellan. Images were taken using a Leica DMi8 automated microscope. To analyze the staining intensity, image analysis was performed using ImageJ software [59].

### 4.11. TUNEL Staining

Paraffin-embedded knee sections were deparaffinized, rehydrated, and stained using the ApopTag^®^ Red in situ apoptosis detection kit from Millipore (Merck Chemicals, Gernsheim, Germany) according to the manufacturer’s instructions. The sections were mounted with Fluoromount + DAPI (00-4959-52, Thermo Fisher, Waltham, MA, USA). Images were taken using a Leica DMi8 automated microscope and image analysis was performed using ImageJ software [59]. The percentage of TUNEL-positive cells was quantified relative to the total number of DAPI-positive cells.

### 4.12. Statistical Analysis

For every experiment, at least 3 individual samples were analyzed as specifically described in Table S1 (see Supplementary Materials). All samples were blinded during evaluation. In the treadmill experiments, the mice were randomly assigned to the control or exercise group. Potential confounders were minimized by performing the treadmill exercise at the same time with the same procedure for all animals. Damaged histological samples were excluded from evaluation. Data are presented as scattered plots as means ± SD with a confidence interval of 95%. Parametric, two-sided (Student *t*-test) tests were performed using GraphPad Prism V.6.0 h (GraphPad Software Inc., La Jolla, CA, USA), with *p* < 0.05 determining the primary level of significance. Exact *p*-values are reported in the figure legends.

## Data Availability

Not applicable.

## Supplementary Materials

The following supporting information can be downloaded at: https://www.mdpi.com/article/10.3390/ijms23010182/s1.
