# Supplementary material for: ER Stress in ERp57 Knockout Knee Joint Chondrocytes Induces Osteoarthritic Cartilage Degradation and Osteophyte Formation
Source: Int J Mol Sci. 2021 Dec 24;23(1):182. doi: 10.3390/ijms23010182 (PMC8745280; doi:10.3390/ijms23010182)
Supplement: Supplementary file 1 [file ijms-23-00182-s001.zip › ijms-1487304-supplementary.pdf]

Supplementary Material

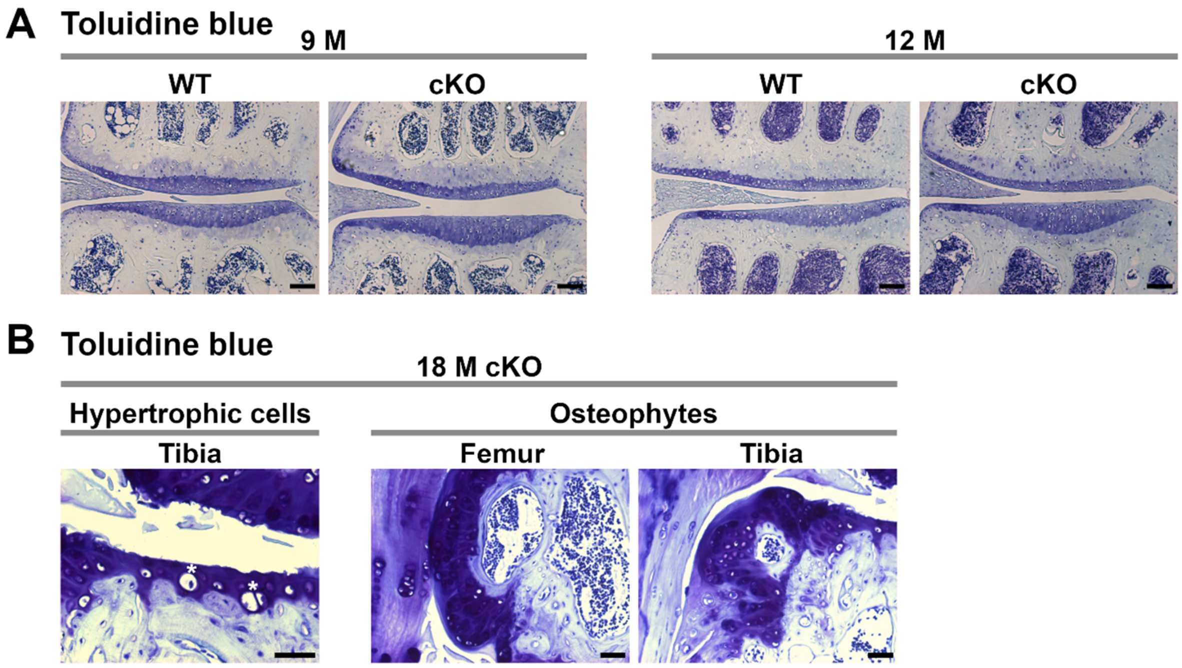

Figure S1: (A) ERp57 cKO mice show no differences in histological analysis of toluidine blue stained knee sections at the age of 9 and 12 months. Scale Bars = 100 µm. (B) Toluidine blue stained knee sections of 18-month-old mice reveal the occurrence of hypertrophic chondrocytes (*) and osteophyte formation in ERp57 cKO mice. Scale bars = 50 µm. See also figure 3 in the main manuscript.

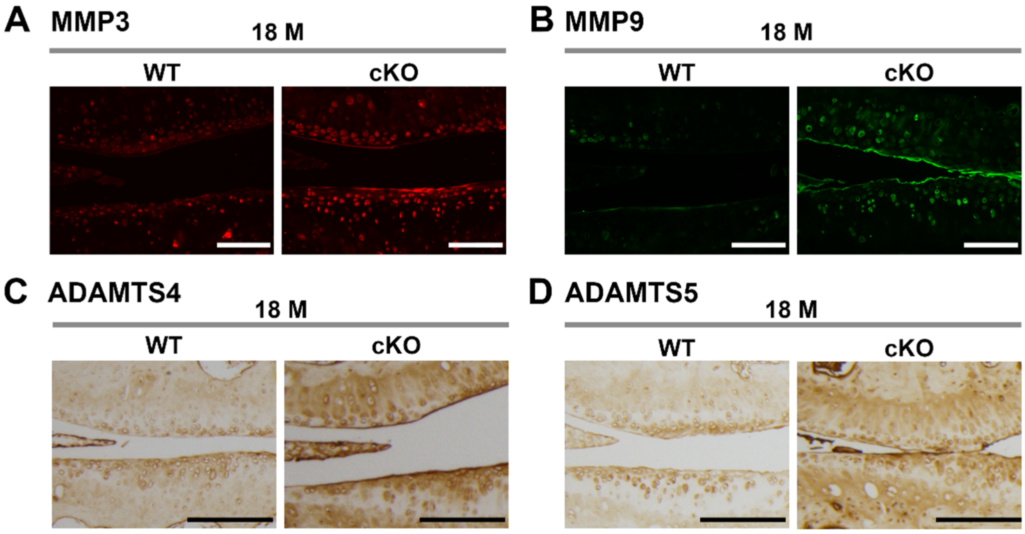

Figure S2: (A), (B) At the age of 18 months ERp57 cKO mice display increased staining of cartilage degrading enzymes MMP3 and MMP9 compared to WT animals. Scale bars = 50 µm. (B), (D) Likewise, staining intensity of the aggrecanases ADAMTS4 and ADAMTS5 are increased in 18 months ERp57 cKO mice compared to WT animals. Scale bars = 100 µm. See quantification in Figure 3C of the main manuscript.

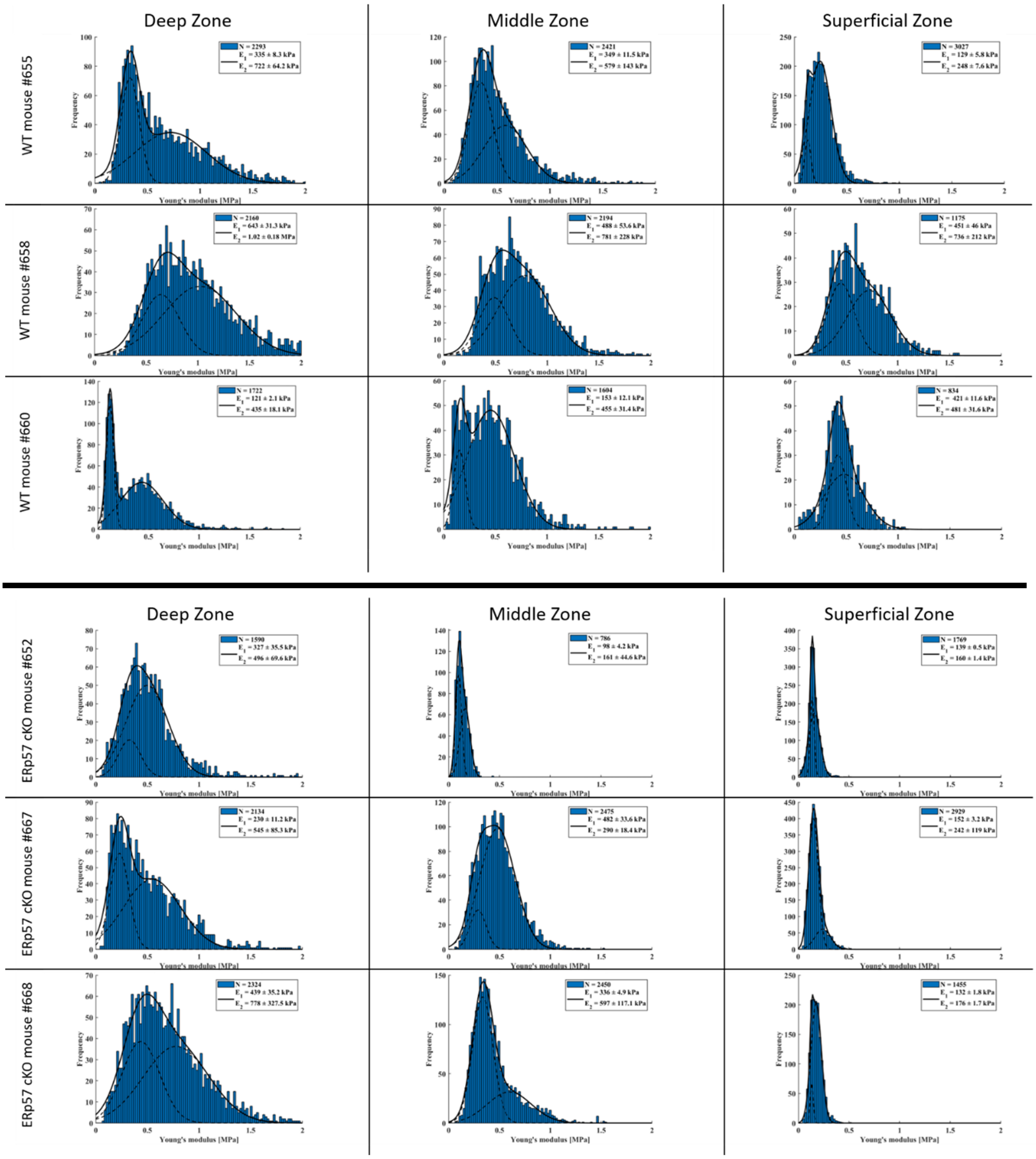

Figure S3: At the age of 14 weeks knee cartilage samples from 3 individual ERp57 cKO and WT animals were analyzed by Indentation-type atomic force microscopy (IT-AFM). Histograms of the Young's moduli reveal bimodal Young's modulus distributions. The low modulus peak depicts the proteoglycan moiety, whereas the second peak represents the collagen fibril network. All values are depicted as peak values ± 2-fold standard deviation of the fitted curve. This corresponds to a confidence interval of 95 %. The individual results of the 14-week-old ERp57 cKO animals display a shift of proteoglycan and collagen peaks towards lower values compared to WT mice. This demonstrates a decreased compressive stiffness in all three zones of the knee joint cartilage under ER stress. N = Number of individual data points, E_1 = Peak of proteoglycan stiffness values, E2 = Peak of collagen stiffness values, kPa = Kilopascal, MPa = Megapascal.

Table S1: Study design and samples

Readout	Method	Figure	Groups	Sample Size	Comments
In *vitro* analysis of ER stress in articular knee cartilage	(Immuno)fluorescence Analysis of BiP, Calnexin, Thioflavin T and Ubiquitin	1A	C28/I2 WT and C28/I2 ERp57KO cells	3 experiments	3 pictures/experiment. Quantification of at least 5 cells per picture.
In *vivo* analysis of ER stress in articular knee cartilage	Transmission electron microscopy	1B	newborn WT and ERp57cKO mice	3 WT and 3 ERp57 cKO mice	3 areas/sample
	BiP staining	1B	14-week-.old WT and ERp57cKO mice	3 WT and 3 ERp57 cKO mice	Analysis of the whole joint area
Stiffness measurments of articular knee cartilage	Atomic Force Microscopy	2	14-week -old WT and ERp57cKO mice	3 WT and 3 ERp57 cKO mice	9 areas/sample, 5625 data points per animal
Osteoarthritis scoring of articular knee cartilage	OARSI scoring of toluidine blue stained sections	3A	9, 12 and 18-month-old WT and ERp57cKO mice	9 M = 5 WT & 4 ERp57 cKO mice<br>12 M = 6 WT & 7 ERp57 cKO mice<br>18 M = 5 WT & 5 ERp57 cKO mice	Analysis of femoral condyle and tibial plateau of the medial and the lateral joint. Highest score of the four quadrants was taken for the analysis
		4A	14-week-old WT and ERp57cKO mice and 14-week-old Treadmill WT and ERp57cKO mice	14 W = 3 WT & 4 ERp57 cKO mice<br>14 W TM = 3 WT & 4 ERp57 cKO mice	
Apoptosis assay of articular knee cartilage	TUNEL assay	3B	9, 12 and 18-month-old WT and ERp57cKO mice	9 M = 5 WT & 4 ERp57 cKO mice<br>12 M = 6 WT & 7 ERp57 cKO mice<br>18 M = 4 WT & 5 ERp57 cKO mice	4 data points/animal. One data point accounts for one quadrant of the joint area.
		4B	14-week-old WT and ERp57cKO mice and 14-week-old Treadmill WT and ERp57cKO mice	14 W = 3 WT & 4 ERp57 cKO mice<br>14 W TM = 3 WT & 4 ERp57 cKO mice	
Analysis of matrix degrading proteases in the articular knee cartilage	Fluorescent staining of MMP3 and MMP9	3C	18-month-old WT and ERp57cKO mice	4 WT & 4 ERp57 cKO mice	4 data points/animal. One data point accounts for one quadrant of the joint area.
	Histological staining of ADAMTS4 and ADAMTS5			5 WT & 5 ERp57 cKO mice	

W = weeks, M = months, TM = treadmill
